# Inter-generational differentials in perceptions of intimate partner violence in Nigeria

**DOI:** 10.1371/journal.pone.0327214

**Published:** 2025-07-15

**Authors:** Treasure C. I. Ntoimo, Beatrice Adeoye, Lorretta F. C. Ntoimo

**Affiliations:** 1 Department of Sociology, Faculty of Social Sciences, Federal University Oye-Ekiti, Nigeria; 2 Department of Demography and Social Statistics, Faculty of Social Sciences, Federal University Oye-Ekiti, Nigeria; India Health Action Trust (IHAT), Uttar Pradesh Technical Support Unit (UP-TSU), INDIA

## Abstract

Despite global efforts, gender-based violence (GBV) remains a problem that affects millions of people, particularly women. The prevalence of GBV in Nigeria has not improved over time; women who experienced physical violence since age 15 increased from 28% in 2008 to 31% in 2018. Intimate partner violence (IPV) constitutes a large proportion of the GBV in Nigeria. Although perceptions of IPV have been studied, differentials in perceptions among the different generations of Nigerians are unknown. People’s perceptions of IPV are influenced by societal shifts and other factors that differ among people of various ages. This research examined inter-generational differentials in the perception of IPV in Nigeria. Data were obtained from the 2021 Nigeria Multiple Indicator Cluster Survey. A sample of 53,706 men and women was analyzed using descriptive statistics, and logistic regression models. The older generations of men and women in Nigeria have significantly better perceptions of IPV than the younger generation, but there is a significant variation at the sub-national level. The observed pattern is worrisome and calls for urgent action by the government to advance more positive perceptions of GBV in Nigeria if the country will make progress in reducing the prevalence of GBV and achieve a violence-free society.

## Introduction

Gender-based violence (GBV) is violence directed against a person because of that person’s gender or violence that affects persons of a particular gender disproportionately [1,2]. The most common form of GBV is intimate partner violence (IPV), violence perpetrated by a sexual partner. IPV occurs in different forms such as physical abuse, sexual abuse, psychological abuse, and stalking. Despite the efforts of global and national organizations to combat GBV, IPV is still a global problem that affects several million people, particularly women [2,3]. It was made worse by COVID-19 as many women were trapped with their abusers due to lockdown and other restrictions on movement [4,5]. Globally, one in every three women, or approximately 736 million women, has experienced intimate partner violence, non-partner sexual violence, or both at least once in their lifetime [6]. This excludes extreme cases of death; 47,000 women were killed by their intimate partners or family members in 2020, amounting to 1 woman killed every 11 minutes [7]. According to the 2018 Nigeria Demographic Health Survey, 36% of ever-married women aged 15–49 in Nigeria have experienced physical, emotional or sexual spousal violence [8]. Of note is that the prevalence of any form of GBV in Nigeria has not improved over time. For instance, the percentage of women who had ever experienced physical violence since age 15 was 28% in 2008, remained the same in 2013 and increased to 31%, ten years after in 2018. Violence perpetrated by a male spouse has also shown no improvement over this same period. The prevalence of physical, sexual, or emotional violence against women by an intimate partner increased from 31% in 2008 and 25% in 2013 to 36% in 2018 [8–10].

IPV is life-threatening with long-lasting adverse consequences on survivors and their families, and high economic costs [2,6]. It can lead to death, physical harm, and long-term emotional and health problems, among others. Marital rape and sexual assault result in unwanted pregnancies, and complications during pregnancy and birth. Although there has been advancement in the fight against IPV it is becoming increasingly apparent that attitudes and views of the problem differ greatly between generations, and affect the perpetration and persistence of the act [11]. The issue at stake is the dearth of thorough knowledge of these generational differences in attitudes toward intimate partner violence in countries such as Nigeria and how they affect the development of successful preventative and intervention plans.

In a time of changing gender roles, societal norms, and communication technology, it is clear that generational viewpoints have a significant impact on how people respond to intimate partner violence. Nevertheless, there is a paucity of empirical research that methodically investigates how people of various ages view and understand intimate partner violence. This lack of empirical evidence makes it more difficult to create customized interventions that successfully involve different generations and deal with the underlying causes of IPV. Most existing studies on the subject of GBV concentrate on intimate partner violence at the societal level and how society justifies it as part of male prestige, illustrating how masculinity prevails to justify violence, examining the perception of community members, women and religious leaders on intimate partner violence, and the determinants of IPV [12–16]. People’s perspectives and reactions to IPV are likely to be influenced by societal shifts, educational and media exposure, and cultural influences, and these would differ among people of different ages. The absence of a thorough examination of these elements across generations hinders attempts to close the generational divide and develop integrated GBV prevention strategies.

There is an urgent need for studies that explore the generational differences in attitudes toward intimate partner violence. Through a thorough comprehension of how these disparities materialize and the identification of the fundamental causes, society may create more persuasive public awareness campaigns, educational programmes, and policy measures that appeal to individuals of all ages. Closing this knowledge gap is critical to establishing a more equal and safe environment for all people, regardless of age. Therefore, this research investigates intergenerational differentials in perceptions of IPV in Nigeria. The specific objective is to examine the variation in perceptions of IPV among younger and older generations of women and men in Nigeria.

### Theoretical framework

The theory of generations by Karl Mannheim, and the social-ecological theory provided the theoretical framework for this research. The theory of generations, also known as the sociology of generations, was developed by Karl Mannheim. In his article “The Sociological Problem of Generations,” Mannheim argued that major events can shape the way people think and act based on when they were born and what they went through during their formative years. [17]. As a result, these shared experiences create social groups or “cohorts” that go on to shape future generations. Some critics argue that this theory is limited to Western ideas and does not take into account other cultures and societies. However, others believe that the globalized nature of modern society means that the theory should be applied more broadly [18,19].

Mannheim defined a generation as a group of people who were born around the same time and experienced a significant historical event during their youth. This event shapes their perspectives and consciousness in life. However, not every generation will have its unique perspective, but it depends on how quickly society changes.

Mannheim believed that social change can happen gradually without big events, but those big events are more likely to happen during times of fast change. He also said that people in the same generation may have different perspectives based on their location, culture, and class. This means that even within a generation, there can be different responses to historical situations which creates “social generations”. Mannheim’s theory of generations explains how important historical, cultural, and political events can shape the beliefs and actions of different groups of people based on their age. For example, in the late 1950s and early 1960s, young people in the United States became more aware of inequalities in society through their involvement in the Civil Rights Movement. This led to a belief that these inequalities needed to be changed through individual and collective action. However, by the later part of the 1960s and into the 1970s, younger generations were less engaged in social movement activity because they were more focused on individual fulfilment rather than questioning societal norms [20].

This theory has been applied to various situations such as how young people’s attitudes toward work and politics were affected during the Great Depression or how young Germans’ political attitudes were shaped by the Nazi regime [21]. It has also helped to explain the changing patterns of civic engagement or why same-sex marriage has become more widely accepted over time [22]. Recent applications of this theory include changes in journalism in mainland China [23], climate change as a social issue [24], the creation of a new generation through the COVID-19 pandemic events [25], and tax morality among young generations [26]. Generally, Mannheim’s theory shows how significant events can impact different generations differently depending on where they are developmentally.

The socio-ecological models were developed to understand how different personal and environmental factors interconnect to influence behavior, and how people interpret and respond to events and situations [27,28]. According to Bronfenbrenner (1977), to understand human development, we need to take into account the entire ecological system in which growth occurs. This includes biological and genetic aspects of the person as well as their immediate physical and social environment (microsystem), interactions among systems within that environment (mesosystems), broader social, political, and economic conditions (exosystem), and general beliefs shared by society (macrosystems).

The socio-ecological models have been used to study and explain diverse human behaviour, including aspects of gender violence, such as attitudes and perceptions of GBV [28–31]. The USA Center for Disease Control and Prevention used the social-ecological model to demonstrate the factors that influence violence and the potential strategies for prevention [32]. Drawing from Bronfenbrenner’s model, the current analysis posits that an individual’s perception of IPV can be influenced by a complex interplay of factors operating at the individual (micro), relationship (meso), community (exo), and societal (macro) levels.

Based on the stated background and theoretical framework, the study seeks to test the following hypotheses: 1) The perception of intimate partner violence does not vary significantly among younger and older generations of men and women in Nigeria, and 2) There is no significant variation in the perceptions of IPV across generations at the sub-national level.

## Methods and materials

### Study design and data source

The study is an analysis of cross-sectional data obtained from the round 6 (MICS6) of the Nigeria Multiple Indicator Cluster Survey (MICS), a nationally representative cross-sectional survey conducted in 2021 [33]. MICS is a survey coordinated and sponsored by the United Nations Children’s Fund (UNICEF) in 120 countries since the 1990s. The survey collects data on different issues relating to the well-being of women, children, men, and households, including attitudes toward domestic violence [34]. The data and details of how the 2021 Nigeria survey was organized are accessible from https://mics.unicef.org/surveys?display=card&f[0]=round:53&f[1]=region:4131&f[2]=datatype:0&f[3]=status:241&f[4]=year:2021

### Study setting

The study area is Nigeria. Nigeria is the most populous country in Africa, with a current projected population of 222 million in 2023 [35]. The country is multi-ethnic, with over 250 ethnic groups. These multiple ethnic groups represent diverse cultures that view gender relations differently. Administratively, Nigeria is made up of thirty-six states and a federal capital territory, Abuja. The thirty-six states and Abuja are grouped into six geopolitical zones, also called regions (North-central, Northeast, Northwest, Southeast, South-south, and Southwest). The grouping is based on states with similar sociocultural orientations and geographical proximity. Nigeria has legislation against violence and is a signatory to international initiatives and agreements against all forms of violence. The country’s constitution is against violence and the inhuman treatment of anybody. The 1999 Constitution of the Federal Republic of Nigeria (as amended), section 34 on rights to dignity of human persons provides that: “No person shall be subjected to torture or to inhuman or degrading treatment. No person shall be held in slavery or servitude and no person shall be required to perform forced or compulsory labour” [36]. Also, there is the Violence Against Persons Prohibition (VAPP) Act of 2015 which among other forms of violence and harmful practices prohibits placing a person in fear of physical injury willfully; deprivation of liberty; forced financial dependence or economic abuse; forced isolation or separation from family and friends; emotional, verbal and psychological abuse; and intimidation [37].

### Study population

The study population for this research is a nationally representative sample of women and men aged 15–49 years who were successfully interviewed in the 2021 Multiple Indicator Cluster Survey in Nigeria.

### Sample size

The weighted analytical sample size for this research is 53,706, comprising 16,571 men aged 15–49 years, and 37,135 women aged 15–49 years. This sample size is derived after dropping all the don’t know(s) and no response from the weighted sample of 38,806 women and 17,347 men in the 2021 Nigeria MICS. The missing values (no response) and don’t know responses for both men’s and women’s data were less than 2.5% in each of the variables included in this analysis. The details are provided in the supporting information (S1. Missing values and don’t know responses).

### Sample technique

The sample design for the 2021 Nigeria MICS aimed to generate statistically reliable estimates of various indicators, nationally, in urban and rural settings, and across the 37 strata encompassing all 36 states and the Federal Capital Territory (FCT). A multi-stage, stratified cluster sampling methodology was employed for the selection of the survey sample. The details on sampling are published elsewhere [33].

### Method of data collection

Five questionnaires were utilized in the survey: 1) a household questionnaire aimed at gathering fundamental demographic data on all de jure household members (regular residents), the household itself, and the residence; 2) a questionnaire for individual women administered within each household targeting all women aged 15–49 years; 3) a questionnaire for individual men in half of the chosen households targeting all men aged 15–49 years; 4) a questionnaire for children under 5 years old, administered to mothers (or caregivers) of all children under 5 residing in the household; and 5) a questionnaire for children aged 5–17 years, administered to the mother (or caregiver) of one child aged 5–17 years randomly selected from the household.

For this research, only the questionnaires for men and women are used. The questionnaire for individual women has the following modules: background, mass media and information communication technology (ICT), financial inclusion, fertility/birth history, desire for last birth, maternal and newborn health, postnatal health checks, contraception, unmet need, for family planning female genital mutilation, attitudes towards domestic violence, victimization, marriage/union, sexual behaviour, and life satisfaction. The questionnaire for men consists of the following: background, mass media and ICT, financial inclusion, fertility, attitudes towards domestic violence, victimization, marriage/union, sexual behaviour and life satisfaction. The data were collected by a trained research team using Computer-Assisted Personal Interviewing (CAPI).

### Variables and measures

#### Dependent variable.

The dependent variable for this research is the perception of intimate partner violence. It was measured with six proxy questions on the justification of wife beating fielded to men and women during the survey. The respondents were asked: sometimes a husband is annoyed or angered by things that his wife does. In your opinion, is a husband justified in hitting or beating his wife in the following situations: 1) if she goes out without telling him 2) if she neglects the children 3) if she argues with him 4) if she refuses to have sex with him 5) if she burns the food and 6) if she sleeps with another man. The response options were Yes (coded 1), No (coded 2), and Don’t Know (coded 8). There is an option for non-response coded 9.

Each question is a variable; thus, the six variables were used to generate a single composite variable of perception of IPV for this research. All the don’t know and no response were dropped from the analysis. The code for the yes response is retained as 1, and no is re-coded as 0. The yes and no response in the six variables were aggregated to generate a single variable with two categories: disapproved IPV (a no response in all six questions) and approved IPV (a yes response in one to six questions). For analysis, the codes were reversed so that the disapproved IPV was coded 1 whereas approved IPV was coded 0.

#### Independent variable.

The key independent variable is the inter-generational differential. Generation describes people within a specific population who have experienced significant events during a particular period. The age of men and women is used as a measure of generation in this study. It is re-categorized to differentiate the younger generation from the older generation using the United Nations definition of youth. The United Nations defines youth as someone aged 15–24 [38]. Following this definition, three categories were created 15–24 (youth), 25–34 (young adults), and 35–49 (older adults).

#### Control variables.

Given that the perception of IPV is not only influenced by a generational gap, other variables identified from existing literature as having a significant influence on the perception of IPV [39–42], and available in the dataset, were included as control variables. The variables included the highest education level measured as no education, primary, junior secondary, senior secondary, and higher/tertiary. Others are the frequency of listening to the radio, and watching television; ownership of a phone; use of a computer; use of the internet; place of residence (rural/urban); region (geo-political zones); household wealth index; level of happiness; life satisfaction; ever experienced sex discrimination; marital status (only for women because it was highly correlated (0.74) with number of children ever had for men); number of children ever children; and ownership of a bank account for women only.

### Methods of data analysis

All the analyses are conducted using Stata 17 for Windows. The characteristics of the study population are presented with frequency and percentage. The variation in perception of IPV by age was examined using descriptive statistics (cross-tabulation with frequency and percentage), and chi-square test of association. Logistic regression models were used to examine if intergenerational differentials predict perceptions of IPV holding other factors constant. All the analyses were conducted separately for men and women, and compared. The sample in the datasets is not self-weighting; therefore, sample weights were used for reporting the results. No-response and don’t know were dropped in all the variables. They were all less than 2.5%. All analyses were two-tailed, and statistical significance was set at p<=0.05 (95% confidence interval).

### Ethical considerations

The survey received ethical approval from the steering committee and a review committee comprised of members from the technical committee for the MICS survey. Verbal consent was acquired from each respondent, and for children aged 15–17 who were individually interviewed, adult consent was procured before the child’s assent. All respondents were told of the voluntary nature of their participation, as well as the confidentiality and anonymity of the information provided. Furthermore, respondents were informed of their right to decline to answer certain questions or to terminate the interview at any point [33].

## Results

### Description of the study population

Table 1 presents the characteristics of the study population focusing on demographic, educational, technological, and socioeconomic factors. The age distribution shows a young population. The majority fall between ages 15 and 24 with 40.7% for men and 37.3% for women. Individuals aged 25 to 34 account for 24.3% of the men and 29.3% of the women population. Men and women aged 35 to 49 make up 35.0% and 33.4%, respectively. Educational levels differ between sexes. Respondents who had no formal education were 15.6% for men and 26.7% for women.

**Table 1:** Background characteristics of the study population.

Characteristic	Male	Female
	Percent (N = 16,571)	Percent (N = 37,135)
**Age**		
15-24	40.7	37.3
25-34	24.3	29.3
35-49	35.0	33.4
**Education**		
None	15.6	26.7
Primary	12.1	13.7
Junior secondary	8.8	8.7
Senior secondary	43.2	36.1
Higher/tertiary	20.3	14.7
**Frequency of listening to the radio**		
Not at all	31.4	49.8
Less than once a week	17.6	15.6
At least once a week	25.2	17.9
Almost every day	25.8	16.7
**Frequency of watching TV**		
Not at all	36.4	47.7
Less than once a week	14.8	9.5
At least once a week	24	15.6
Almost every day	24.8	27.1
**Own a mobile phone**		
Yes	74.2	58.8
No	25.8	41.2
**Ever used a computer or a tablet**		
Yes	22.1	13.6
No	77.9	86.4
**Ever used internet**		
Yes	35.9	20.4
No	64.1	79.6
**Place of residence**		
Urban	44.8	45.7
Rural	55.2	54.3
**Estimation of overall happiness**		
Very happy	27.7	42.2
Somewhat happy	42.7	36.9
Neither happy nor unhappy	16.9	13.9
Somewhat unhappy	8.2	4.9
Very unhappy	4.6	2.2
**Life satisfaction in comparison with last year**		
Improved	55.2	65.1
More or less the same	31.5	25
Worsened	13.3	10
**Life satisfaction expectation one year from now**		
Better	82.1	92.4
More or less the same	14.2	6.3
Worse	3.7	1.4
**Ethnic origin**		
Hausa	27.8	27.6
Igbo	14.4	15
Yoruba	17.3	17.9
Fulani	4.5	5
Kanuri	2	1.6
Ijaw	1.4	1.3
Tiv	2.5	2.4
Ibibio	2	2
Edo	1.3	1.3
Others	26.9	25.8
**Ever had a child**		
Yes	44	65.7
No	56	34.3
**Currently married or living with a woman/man**		
Yes, currently married	41.6	56.7
Yes, living with a partner	3	6
No, not in union	55.5	37.3
**Household Wealth index quintile**		
Poorest	17.4	17.8
Second	18.5	18.8
Middle	19.4	19.5
Fourth	22.2	21.4
Richest	22.6	22.6
**In the past 12 months, felt discriminated: Sex**		
Yes	5	6
No	95	94
**Owns a bank account**		
Yes		35.7
No		64.3
**Geopolitical zone**		
North-central	15.0	15.0
Northeast	13.9	13.0
Northwest	24.8	25.3
Southeast	11.4	11.4
South-south	15.2	14.8
Southwest	19.7	20.5

Regarding media exposure, specifically listening to the radio, 31.4% of men and 49.8% of women say they do not listen at all, and 47.7% of women and 36.4% of men never watched television. Technology use reveals disparities in access and consumption. More men (74.2%) own a mobile phone, compared to 58.8% of women. Computer or tablet use is reported by 22.1% of men and 13.6% of women. Men are more likely to use the internet than women, with 35.9% against 20.4%. In terms of happiness, 27.7% of men and 42.2% of women consider themselves very happy. For the level of life satisfaction, 55.2% of men and 65.1% of women reported higher levels of life satisfaction than the previous year.

According to family characteristics, 44% of men and 65.7% of women have children. Marriage and relationship status show that 41.6% of men and 56.7% of women are currently married. The household wealth index is evenly distributed across quintiles. Five percent of men and six percent of women, respectively, reported experiencing sex discrimination in the previous year, and 35.7% of women reported owning bank accounts. The distribution of the population between urban and rural areas is nearly equal. The population is dispersed over several geopolitical regions.

### Perception of IPV

**Fig 1:**
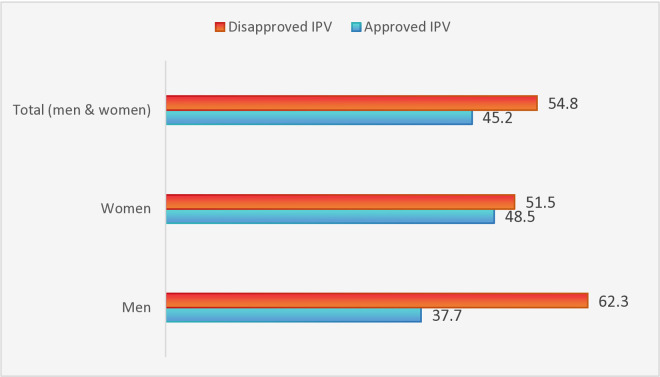
Percentage distribution of perceptions of IPV.

The perception of IPV by the respondents is presented in Fig 1. The perception of IPV among the study population reveals notable differences between men and women. More than half of the men disapproved of IPV, and slightly above half of the women disapproved of IPV. When combining the data for both men and women, the overall perception of IPV among the total population of 53,706 respondents reveals that 54.8% of the entire population disapproved of IPV.

### Intergenerational variation in the perception of IPV

The result of the analysis that examined the variation in perceptions of IPV among younger and older generations of women and men in Nigeria is presented in Table 2. The distribution of perception of IPV by generational differentials is presented for both men and women with the result of the chi-square test of association. Among the men, more than 30% in each age category approved IPV with the largest proportion being among the younger generation aged 15–24 years. Disapproval of IPV was more prevalent among older men aged 35–49 compared to the young adult and younger generations. The relationship between age (generational differentials) and perception of IPV among men is statistically significant. Unlike the men, almost an equal proportion of the three age groups of women approved of IPV, with the largest among the young adult generation aged 25–34 years where about half of them approved of IPV (49.78%). Disapproval of IPV shows a similar pattern to approval of IPV, but the younger women aged 15–24 years had a slightly higher majority who disapproved of IPV compared to the other generations of women. The relationship for women is also statistically significant.

**Table 2:** Variation in perceptions of IPV by generation.

Generation	Men		Women	
	Approved IPV N(%)	Disapproved IPV N(%)	Approved IPV N(%)	Disapproved IPV N(%)
15-24 (Youth)	2,767(41.01)	3,981(58.99)	6,632(47.92)	7,207(52.08)
25-34 (young adult)	1,566(38.93)	2,456(61.07)	5,416(49.78)	5,463(50.22)
35-49 (Older)	1,921(33.12)	3,880(66.88)	5,969(48.07)	6,447(51.93)
Test statistic	Pearson chi2(2) =77.9619 p < 0.001		Pearson chi2(2) = 24.8694 p < 0.001	

The result of the unadjusted logistic regression model predicting the relationship between inter-generational differential (age), and perception of IPV for both men and women is presented in Table 3. The result shows only age as a predictor of perception of IPV, without controlling the effect of any other variable.

**Table 3:** Logistic regression predicting perceptions of IPV by age.

Variable	Men		Women	
	OR	95% CI	OR	95% CI
**Age**				
35-49 (older) (RC)	1		1	
25-34 (young adult)	0.84*	0.77-0.91	0.94*	0.89-0.99
15-24 (younger)	0.72*	0.67-0.78	1.06*	1.01-1.12

Note: *p < 0.05; OR is odds ratio; CI is confidence interval; RC is reference category.

Younger and young adult generation men were significantly less likely to disapprove of IPV compared to the older generation. The odds of disapproving IPV were significantly lower among young adult generation women aged 25–34 years compared to the older generation. In contrast, younger women, aged 15–24 years were significantly more likely to disapprove of IPV compared to the older generation women.

When the effect of other variables is controlled (Table 4), inter-generational differentials (age) remained a significant predictor of perception of IPV. Holding other factors constant, the odds of IPV disapproval remained significantly lower among young adults and younger men compared to older men. For the women, when the effect of other variables is taken into account, the odds of IPV disapproval for the young adult generation remained significantly lower than in older women. The odds for younger women became significantly lower when other factors were adjusted. The younger women became less likely than the older generation to disapprove of IPV.

**Table 4:** Logistic regression models predicting the odds of IPV disapproval.

Variable	Male		Female	
	aOR	95% CI	aOR	95% CI
**Age**				
35-49 (older) (RC)				
25-34 (young adult)	0.82*	0.73 - 0.92	0.90*	0.84 - 0.96
15-24 (Younger)	0.88*	0.78 - 1.00	0.89*	0.82 - 0.97
**Education**				
None(RC)				
Primary	0.97	0.85 - 1.11	1.06	0.97 - 1.14
Junior secondary	0.98	0.84 - 1.14	1.05	0.95 - 1.15
Senior secondary	1.01	0.89 - 1.15	1.11*	1.02 - 1.20
Higher/tertiary	1.12	0.95 - 1.32	1.70*	1.51 - 1.90
**Frequency of listening to the radio**				
Not at all(RC)				
Less than once a week	1.10	0.99 - 1.23	0.99	0.92 - 1.06
At least once a week	1.15*	1.03 - 1.28	0.86*	0.81 - 0.93
Almost every day	1.23*	1.09 - 1.37	0.83*	0.77 - 0.90
**Frequency of watching TV**				
Not at all(RC)				
Less than once a week	0.87*	0.78 - 0.98	1.12*	1.03 - 1.23
At least once a week	0.77*	0.69 - 0.86	1.02	0.95 - 1.11
Almost every day	0.89	0.78 - 1.00	0.96	0.89 - 1.04
**Owns a mobile phone**				
Yes(RC)				
No	0.91*	0.83 - 0.99	1.09*	1.03 - 1.15
**Ever used a computer or a tablet**				
Yes(RC)				
No	0.72*	0.63 - 0.81	0.91	0.83 - 1.00
**Ever used internet**				
Yes(RC)				
No	0.66*	0.60 - 0.73	0.79*	0.73 - 0.85
**Place of residence**				
Urban(RC)				
Rural	0.97	0.88 - 1.07	0.81*	0.76 - 0.86
**Estimation of overall happiness**				
Very happy(RC)				
Somewhat happy	1.11*	1.02 - 1.22	1.06*	1.00 - 1.12
Neither happy nor unhappy	0.96	0.85 - 1.08	0.98	0.91 - 1.06
Somewhat unhappy	0.80*	0.69 - 0.93	0.92	0.82 - 1.03
Very unhappy	0.96	0.79 - 1.17	1.14	0.97 - 1.34
**Life satisfaction in comparison with last year**				
Improved(RC)				
More or less the same	1.24*	1.13 - 1.36	1.17*	1.11 - 1.24
Worsened	1.84*	1.61 - 2.10	0.87*	0.80 - 0.95
**Life satisfaction expectation one year from now**				
Better(RC)				
More or less the same	0.84*	0.75 - 0.93	0.95	0.87 - 1.05
Worse	0.52*	0.43 - 0.64	1.42*	1.16 - 1.73
**Ethnic origin**				
Hausa(RC)				
Igbo	0.74*	0.58 - 0.95	1.24*	1.07 - 1.45
Yoruba	1.31*	1.06 - 1.61	1.59*	1.39 - 1.82
Fulani	1.29*	1.07 - 1.55	1.06	0.95 - 1.19
Kanuri	0.75*	0.57 - 0.97	0.89	0.74 - 1.07
Ijaw	0.83	0.60 - 1.16	1.13	0.91 - 1.42
Tiv	0.76*	0.58 - 0.98	1.35*	1.14 - 1.60
Ibibio	0.64*	0.47 - 0.87	0.91	0.75 - 1.11
Edo	2.00*	1.34 - 2.98	1.49*	1.19 - 1.88
Others	0.78*	0.67 - 0.89	1.03	0.94 - 1.13
**Number of children ever had**	1.01	1.00 - 1.03	0.98**	0.97 - 0.99
**Marital Status**				
Currently married(RC)				
Living with a partner			0.79*	0.71 - 0.87
Not in a union			1.24*	1.16 - 1.32
**Household wealth index quintile**				
Poorest(RC)				
Second	1.05	0.93 - 1.17	0.98	0.91 - 1.05
Middle	1.15*	1.02 - 1.31	1.16*	1.06 - 1.26
Fourth	1.36*	1.18 - 1.58	1.30*	1.18 - 1.43
Richest	1.65*	1.39 - 1.96	1.58*	1.41 - 1.77
**In the past 12 months, felt discriminated: Sex**				
Yes(RC)				
No	1.91*	1.63 - 2.23	1.37*	1.24 - 1.51
**Geopolitical zone of residence**				
North-central(RC)				
Northeast	1.51*	1.31 - 1.75	1.23*	1.12 - 1.35
Northwest	1.75*	1.49 - 2.04	0.70*	0.63 - 0.77
Southeast	1.10	0.86 - 1.41	0.88	0.76 - 1.03
South-south	0.87	0.75 - 1.00	0.67*	0.61 - 0.73
Southwest	3.17*	2.66 - 3.79	2.50*	2.23 - 2.80
**Own a bank account**				
Yes(RC)				
No			0.99	0.93 - 1.06

Note: *p < 0.05; **p < 0.01; ***p < 0.001; aOR - adjusted odds ratio; CI – confidence interval; RC – reference category.

Some control variables were statistically significant predictors of IPV disapproval. The significant variables included education, number of children, marital status, and place of residence for women; ever used a computer or tablet for men only; and media exposure (frequency of listening to the radio and watching television), ownership of a mobile phone, use of the internet, estimation of overall happiness, life satisfaction, ethnic origin, household wealth index, sex discrimination, and region of residence for both men and women.

### Differentials in perception across generations at the sub-national level

A model that has an interaction of the age groups and the geo-political zone was estimated to examine variations in perceptions of IPV across generations at the sub-national level (Table 5). Holding other factors constant, the odds of IPV disapproval are significantly higher among the older generation (35–49 years old) men in the Northeast, Northwest, and Southwest compared to their counterparts in the North-central. Among women, there are lower odds of IPV disapproval among older generation women in the Northwest compared to the older generation women in the North-central. This result contrasts with the higher odds among men in the Northwest. The odds of IPV disapproval were significantly lower among older women in the Southeast and South-south. Like the men, older women resident in the Southwest were significantly more likely to disapprove of IPV relative to the older generation women in the North-central.

**Table 5:** The Odds of Intergenerational Perception of IPV in Geo-Political Zones.

Variable	Men		Women	
	aOR	95% CI	aOR	95% CI
35-49 x North-central (RC)	1.00	1.00 - 1.00	1.00	1.00 - 1.00
35-49 x Northeast	1.41**	1.11 - 1.79	1.07	0.92 - 1.26
35-49 x Northwest	1.96***	1.55 - 2.47	0.65***	0.56 - 0.75
35-49 x Southeast	0.91	0.67 - 1.23	0.73**	0.60 - 0.88
35-49 x South-south	0.96	0.78 - 1.20	0.65***	0.57 - 0.76
35-49 x Southwest	2.22***	1.74 - 2.83	1.98***	1.69 - 2.32
25-34 x North-central	0.90	0.71 - 1.13	0.79**	0.68 - 0.91
25-34 x Northeast	1.23	0.96 - 1.57	0.94	0.80 - 1.09
25-34 x Northwest	1.27	1.00 - 1.60	0.55***	0.47 - 0.63
25-34 x Southeast	0.83	0.58 - 1.17	0.83	0.68 - 1.02
25-34 x South-south	0.61***	0.47 - 0.78	0.49***	0.42 - 0.58
25-34 x Southwest	2.15***	1.62 - 2.86	2.29***	1.93 - 2.71
15-24 x North-central	0.72**	0.58 - 0.89	0.74***	0.64 - 0.86
15-24 x Northeast	1.20	0.96 - 1.51	1.05	0.90 - 1.22
15-24 x Northwest	1.33*	1.06 - 1.67	0.56***	0.48 - 0.65
15-24 x Southeast	0.98	0.72 - 1.33	0.69***	0.56 - 0.84
15-24 x South-south	0.61***	0.48 - 0.77	0.52***	0.44 - 0.60
15-24 x Southwest	4.22***	3.17 - 5.62	2.10***	1.76 - 2.51

Note: *p < 0.05; **p < 0.01; ***p < 0.001; aOR - adjusted odds ratio; CI – confidence interval; RC – reference category.

In comparison to the older generation men in the North-central, only the young adult generation (25–34 years old) men residents in the Southwest are significantly more likely to disapprove of IPV. The young adult generation men in the South-south were significantly less likely to disapprove of IPV than older men in the North-central. Among the mid-generation women, the odds of IPV disapproval were significantly lower in the North-central, Northwest, and South-south, but higher in the Southwest compared to older women in the North-central.

The odds of IPV disapproval were significantly lower among the younger generation (15–24 years old) men in the North-central, and South-south; whereas significantly higher odds were observed in the Northwest and Southwest. Among the younger generation women, the odds of IPV disapproval were significantly lower in the North-central, Northwest, Southeast, and South-south, but higher in the Southwest.

## Discussion of the findings

The general objective of this research is to investigate intergenerational differentials in perceptions of IPV in Nigeria using age groups as a measure of generational differences. The hypotheses that there is no variation in perceptions of IPV among younger and older generations of women and men in Nigeria, and at the sub-national level were tested The result shows that even though the majority of both men and women exhibit disapproval of IPV, there is still a substantial proportion who approve of IPV. It is worth noting that more women approved of IPV compared to men; and except in the Southwest region, the odds of disapproving of IPV were significantly lower among women of all age groups. This result may be because men are more likely than women to give socially desirable responses for social reasons, being the usual perpetrators of IPV. Women in Nigeria as in many other countries, are the usual victims of IPV, and tradition and gender norms in many Nigerian settings view IPV as an acceptable expression of masculinity and a means to correct women and children [43]. Having imbibed and internalised these traditions and norms, some women may see nothing wrong with IPV. More women than men in the study population have no education, and no exposure to the media, and the internet. These disadvantages limit women’s exposure to knowledge that could empower them to challenge the traditions and norms that approve IPV. These findings highlight the pressing need for more comprehensive education and awareness initiatives aimed at improving the understanding of IPV across the entire population, with a particular focus on addressing and altering perceptions among women. A study of young adults in the USA found more supportive attitude towards IPV among women [44]. In contrast to this finding, a previous study conducted in Ethiopia revealed that men expressed more support for GBV when compared to women [41].

Regarding generational differences, the younger generations (25–34 and 15–24 age groups) are more likely to approve of IPV than the older generations (35–49 age group), of both men and women. Typically, older adults hold more conservative views, which may align with tradition and potentially approving IPV in certain cultural contexts [45]. However, this study shows the opposite, possibly because younger people have less life experience and exposure to advocacy and education on IPV. This result may also be linked to the increasing prevalence of violence against women across the globe, and technology-facilitated gender-based violence, which is common among Generation Z and Millennials [46]. The younger generations are more likely to see violence against women as normal due to their higher exposure to these events through social media.

According to Mannheim in the theory of generations, individuals are heavily influenced by the historical events they experience during their youth [17]. This result is suggestive of slow progress in challenging patriarchal gender norms in Nigeria that support male superiority, gender inequality, and GBV [40,47–49]. These norms persist in the private and public spheres in the country despite expanded opportunities for education, media campaigns and legislation against violence. Nigeria has legislation against all forms of gender-based violence such as the Violence Against Persons Prohibition (VAPP) Act of 2015 [37], and the 1999 Constitution of the Federal Republic of Nigeria (as amended), section 34 [36].

However, there are some variations at the sub-national level. Young adults and younger men and women in the Southwest significantly disapproved of IPV compared to the older generation. The inclination among the younger generations, and indeed all the generations in the Southwest to disapprove of IPV may be because the Southwest is the most urbanised region in Nigeria with a large population of residents who are migrants and educated. The southwest is home to Lagos, the most urbanised city in Nigeria. The advantage of urbanism and education may have contributed to the better perception of IPV in that region [50]. Also, the region has the lowest prevalence of IPV (20.1%) in Nigeria [8].

In the Northwest, all generations of men disapproved of IPV, although it was not statistically significant for the mid-generation men. On the contrary, no generation of women in the region disapproved of IPV. A similar pattern was observed in the Northeast, but the only significant result was among older generation men. The tendency to disapprove of IPV by men in the Northwest may be because of the relatively low prevalence of IPV in that region, the second lowest in Nigeria after the Southwest [8]. The gender gap in the proportion who are educated in these two regions is wide. Men who have no education in the Northwest are 30% compared to 55% for women, and in the Northeast, it is 37% for men and 54% for women [33]. These two regions have a predominantly Muslim population, polygyny is prevalent and a large proportion of women are financially dependent on their spouses [8,33]. Low level of education, polygyny and financial dependency increases the risk of experiencing IPV by women in Nigeria and other countries in sub-Saharan Africa [51–55] and may contribute to women’s tendency to submit to the normalisation of IPV.

The odds of IPV disapproval for all generations of both men and women in the North-central were significantly lower. This may be attributed to the North-central region having the highest prevalence of intimate partner violence (physical, sexual, and emotional) in Nigeria, with one state in the region reporting a prevalence rate of 75% [8]. The ubiquitous nature of IPV in this region may have contributed to the significant approval of IPV among both men and women. Exposure to violence is shown to be significantly related to a supportive attitude to IPV [44]. It is also likely that prevailing marriage norms such as forced marriage, arranged marriage and concubinage in the region may also encourage acceptance of IPV as normal [56]

Like the North-central, all the generations of men and women in the South-south significantly approved IPV except among the older generation of men, where the odds were statistically insignificant. Also, in the Southeast, no generation of men and women disapproved of IPV but it was only significant among younger and older generation women. The southern regions of Nigeria have better socio-economic development indicators, such as a higher literacy rate and a higher proportion of women in the labour force, among others [8]. However, the prevalence of IPV in the Southeast and South-south as well as the perceptions of IPV as shown in this study, differ significantly from the Southwest. The prevalence of IPV in these two southern regions is double the rate in the southwest [8]. This may have contributed to the approval of IPV in the Southeast and South-south. Also, the Southeast and South-south have a predominant Christian population and have more conservative cultures of marriage and divorce than the Southwest. Acceptance of IPV is more likely in the regions, at least to protect a union from dissolution through divorce [57].

The study results also identified several factors such as secondary and tertiary education for women, exposure to the media (radio and television), ownership of a mobile phone, and life satisfaction and use of the internet, among others, that significantly predict IPV disapproval. This finding indicates that the perception of IPV might be mediated by socioeconomic factors. These findings are consistent with a previous study in Nigeria and other countries, which observed that individual and contextual factors are significantly related to perceptions of GBV [40,45,58]. Additionally, these findings align with the social-ecological theory’s propositions, which suggest that an individual’s perception of IPV is influenced by a complex interplay of factors at the individual (micro), relationship (meso), community (exo), and societal (macro) levels [27,28].

Based on the findings, the following recommendations are made for policy and programmes aimed at improving perceptions of IPV to a more positive one, thereby reducing the prevalence of IPV in Nigeria: 1) Although both men and women should be targeted in interventions, women would need specific attention to change the orientation that has been imbibed by them that IPV is acceptable. Unless women’s perception of IPV significantly improves, the high prevalence of IPV will persist in Nigeria. 2) That the younger generation is significantly less likely to disapprove of IPV in all the regions except the Southwest is worrisome. One would expect the younger people to be less conservative about IPV given the many campaigns against GBV. This finding calls for proactive measures to identify particular reasons behind the way younger people see GBV and tackle them from different sectors such as including gender and respect for women in the school curriculum; and social media campaigns against GBV tailored to younger generations. 3) Approval of IPV among young people and women is indicative of the persistence of gender social norms that undervalue women [59]. Engaging the communities in initiatives that reinforce disapproval of GBV, gender equality, and equity can create a powerful shift in attitudes, fostering a more respectful and inclusive society.

Further studies that incorporate qualitative research are recommended to uncover the reasons behind the perceptions held by the respondents. This study has some limitations. The measure of generation is limited by the data source. Typically, a generation refers to the average period, usually about 20–30 years, during which children are born, grow up, become adults, and start having their children. Also, the data is cross-sectional, thus, the generalization of the results is limited to the particular time when the data were collected. The perceptions across the various groups may have changed. Also, the proxy questions used to assess perceptions of IPV only capture physical violence, other forms of IPV such as emotional, and sexual violence were not included. Nevertheless, the result provides useful insight into people’s perceptions of intimate partner violence against women. Given the subjective nature of the responses to the proxy questions that are used to measure perceptions of IPV, social desirability bias may not be ruled out. Dropping missing values and don’t know responses (although these are less than 2.5%) may have eliminated respondents whose perceptions of IPV may have affected the overall results.

## Conclusions

Perceptions of intimate partner violence in Nigeria vary among the different generations of men and women in Nigeria. Older generations hold a more positive perception of IPV than the younger generations but there are variations at the sub-national level. The observed pattern calls for urgent actions to advance better perceptions of IPV for Nigeria to make substantial progress towards reducing the prevalence of GBV and achieving the sustainable development goal of a violence-free society.

## Supporting information

S1 Missing values and don’t know responses. (DOCX)
