# Supplementary material for: Inter-generational differentials in perceptions of intimate partner violence in Nigeria
Source: PLoS One. 2025 Jul 15;20(7):e0327214. doi: 10.1371/journal.pone.0327214 (PMC12262849; doi:10.1371/journal.pone.0327214)
Supplement: S1 — (DOCX) [file pone.0327214.s001.docx]

**SUPPORTING INFORMATION: Missing values and don’t know responses**

Weighted frequencies showing the don’t know responses and missing values (no response)

tab1 MDV1A MDV1B MDV1C MDV1D MDV1E MDV1F MWAGE mwelevel MMT2 MMT3 MMT11 MMT4 MMT9 HH6 zone MLS1 MLS3 MLS4 MWM14 MCM1 MMA1 windex5 MVT22B [iw=wt]

**MEN**

-> tabulation of MDV1A

If she goes |

out with out |

telling |

husband: wife |

beating |

justified | Freq. Percent Cum.

---------------+-----------------------------------

1. YES |1,260.90648 7.27 7.27

2. NO | 15,863.124 91.45 98.71

8. DK | 210.638919 1.21 99.93

9. NO RESPONSE | 12.3310639 0.07 100.00

---------------+-----------------------------------

Total | 17,347 100.00

-> tabulation of MDV1B

If she |

neglects the |

children: wife |

beating |

justified | Freq. Percent Cum.

---------------+-----------------------------------

1. YES | 1,715.6657 9.89 9.89

2. NO | 15,424.531 88.92 98.81

8. DK | 196.824855 1.13 99.94

9. NO RESPONSE | 9.97890651 0.06 100.00

---------------+-----------------------------------

Total | 17,347 100.00

-> tabulation of MDV1C

If she argues |

with husband: |

wife beating |

justified | Freq. Percent Cum.

---------------+-----------------------------------

1. YES | 1,792.2752 10.33 10.33

2. NO |15,337.9359 88.42 98.75

8. DK | 202.909095 1.17 99.92

9. NO RESPONSE | 13.8798587 0.08 100.00

---------------+-----------------------------------

Total | 17,347 100.00

-> tabulation of MDV1D

If she refuses |

sex with |

husband: wife |

beating |

justified | Freq. Percent Cum.

---------------+-----------------------------------

1. YES | 2,519.9523 14.53 14.53

2. NO | 14,392.457 82.97 97.49

8. DK |371.2066962 2.14 99.63

9. NO RESPONSE | 63.3844024 0.37 100.00

---------------+-----------------------------------

Total | 17,347 100.00

-> tabulation of MDV1E

If she burns |

the food: wife |

beating |

justified | Freq. Percent Cum.

---------------+-----------------------------------

1. YES | 1,409.3444 8.12 8.12

2. NO | 15,725.516 90.65 98.78

8. DK | 199.183651 1.15 99.93

9. NO RESPONSE |12.95548445 0.07 100.00

---------------+-----------------------------------

Total | 17,347 100.00

-> tabulation of MDV1F

If she sleeps |

with another |

man | Freq. Percent Cum.

---------------+-----------------------------------

1. YES | 5,561.948 32.06 32.06

2. NO | 11,368.823 65.54 97.60

8. DK |347.9450594 2.01 99.61

9. NO RESPONSE | 68.2839261 0.39 100.00

---------------+-----------------------------------

Total | 17,347 100.00

-> tabulation of MWAGE

Age | Freq. Percent Cum.

------------+-----------------------------------

1. 15-19 | 4,357.8275 25.12 25.12

2. 20-24 | 2,914.2639 16.80 41.92

3. 25-29 | 2,252.2368 12.98 54.90

4. 30-34 |1,905.31752 10.98 65.89

5. 35-39 | 2,165.2288 12.48 78.37

6. 40-44 | 2,016.4116 11.62 89.99

7. 45-49 | 1,735.7139 10.01 100.00

------------+-----------------------------------

Total | 17,347 100.00

-> tabulation of mwelevel

Education | Freq. Percent Cum.

--------------------+-----------------------------------

0. None | 2,719.1155 15.67 15.67

1. Primary | 2,075.2052 11.96 27.64

2. Junior secondary | 1,571.796 9.06 36.70

3. Senior secondary |7,528.41774 43.40 80.10

4. Higher/tertiary | 3,450.1776 19.89 99.99

9. Missing/DK | 2.28803435 0.01 100.00

--------------------+-----------------------------------

Total | 17,347 100.00

-> tabulation of MMT2

Frequency of listening |

to the radio | Freq. Percent Cum.

-------------------------+-----------------------------------

0. NOT AT ALL | 5,467.6273 31.52 31.52

1. LESS THAN ONCE A WEEK | 3,057.7314 17.63 49.15

2. AT LEAST ONCE A WEEK | 4,367.7244 25.18 74.32

3. ALMOST EVERY DAY | 4,442.6928 25.61 99.94

9. NO RESPONSE | 11.2241167 0.06 100.00

-------------------------+-----------------------------------

Total | 17,347 100.00

-> tabulation of MMT3

Frequency of watching TV | Freq. Percent Cum.

-------------------------+-----------------------------------

0. NOT AT ALL | 6,280.8941 36.21 36.21

1. LESS THAN ONCE A WEEK | 2,566.8138 14.80 51.00

2. AT LEAST ONCE A WEEK | 4,148.2131 23.91 74.92

3. ALMOST EVERY DAY | 4,339.9452 25.02 99.94

9. NO RESPONSE | 11.1337731 0.06 100.00

-------------------------+-----------------------------------

Total | 17,347 100.00

-> tabulation of MMT11

Own a mobile |

phone | Freq. Percent Cum.

---------------+-----------------------------------

1. YES | 12,734.858 73.41 73.41

2. NO | 4,601.4552 26.53 99.94

9. NO RESPONSE | 10.6869689 0.06 100.00

---------------+-----------------------------------

Total | 17,347 100.00

-> tabulation of MMT4

Ever used a |

computer or a |

tablet | Freq. Percent Cum.

---------------+-----------------------------------

1. YES | 3,775.2798 21.76 21.76

2. NO | 13,546.047 78.09 99.85

9. NO RESPONSE | 25.6736855 0.15 100.00

---------------+-----------------------------------

Total | 17,347 100.00

-> tabulation of MMT9

Ever used |

internet | Freq. Percent Cum.

---------------+-----------------------------------

1. YES | 5,536.8307 35.33 35.33

2. NO | 10,087.914 64.38 99.71

9. NO RESPONSE | 45.585625 0.29 100.00

---------------+-----------------------------------

Total | 15,670.331 100.00

-> tabulation of HH6

Area | Freq. Percent Cum.

------------+-----------------------------------

1. URBAN | 7,783.507 44.87 44.87

2. RURAL | 9,563.4931 55.13 100.00

------------+-----------------------------------

Total | 17,347 100.00

-> tabulation of zone

Geopolitical |

zone | Freq. Percent Cum.

-----------------+-----------------------------------

1. North Central | 2,608.1921 15.04 15.04

2. North East | 2,378.2222 13.71 28.75

3. North West | 4,473.7308 25.79 54.53

4. South East | 1,933.9033 11.15 65.68

5. South South | 2,563.4172 14.78 80.46

6. South West | 3,389.5345 19.54 100.00

-----------------+-----------------------------------

Total | 17,347 100.00

-> tabulation of MLS1

Estimation of overall |

happiness | Freq. Percent Cum.

-----------------------------+-----------------------------------

1. VERY HAPPY | 4,788.0929 27.60 27.60

2. SOMEWHAT HAPPY | 7,409.5346 42.71 70.32

3. NEITHER HAPPY NOR UNHAPPY | 2,940.0584 16.95 87.26

4. SOMEWHAT UNHAPPY | 1,414.6414 8.15 95.42

5. VERY UNHAPPY | 787.969275 4.54 99.96

9. NO RESPONSE |6.703369908 0.04 100.00

-----------------------------+-----------------------------------

Total | 17,347 100.00

-> tabulation of MLS3

Life satisfaction in |

comparison with last |

year | Freq. Percent Cum.

-------------------------+-----------------------------------

1. IMPROVED | 9,543.5662 55.02 55.02

2. MORE OR LESS THE SAME | 5,508.6742 31.76 86.77

3. WORSENED | 2,276.5824 13.12 99.90

9. NO RESPONSE | 18.177302 0.10 100.00

-------------------------+-----------------------------------

Total | 17,347 100.00

-> tabulation of MLS4

Life satisfaction |

expectation one year |

from now | Freq. Percent Cum.

-------------------------+-----------------------------------

1. BETTER | 14,170.562 81.69 81.69

2. MORE OR LESS THE SAME | 2,531.5363 14.59 96.28

3. WORSE | 623.665827 3.60 99.88

9. NO RESPONSE | 21.235927 0.12 100.00

-------------------------+-----------------------------------

Total | 17,347 100.00

-> tabulation of MWM14

Native language of |

the Respondent | Freq. Percent Cum.

-------------------+-----------------------------------

11. HAUSA |4,964.37816 28.62 28.62

12. IGBO | 2,452.4895 14.14 42.76

13. YORUBA | 2,988.6263 17.23 59.98

14. FULANI | 785.418071 4.53 64.51

15. KANURI | 337.168785 1.94 66.46

16. IJAW | 243.532571 1.40 67.86

17. TIV | 407.204586 2.35 70.21

18. IBIBIO | 336.824846 1.94 72.15

19. EDO | 211.162379 1.22 73.37

96. OTHER LANGUAGE | 4,620.1948 26.63 100.00

-------------------+-----------------------------------

Total | 17,347 100.00

-> tabulation of MCM1

Ever fathered |

a child | Freq. Percent Cum.

---------------+-----------------------------------

1. YES | 7,436.6305 42.87 42.87

2. NO |9,865.17326 56.87 99.74

8. DK | 37.9258664 0.22 99.96

9. NO RESPONSE | 7.27040541 0.04 100.00

---------------+-----------------------------------

Total | 17,347 100.00

-> tabulation of MMA1

Currently married or living |

with a woman | Freq. Percent Cum.

------------------------------+-----------------------------------

1. YES, CURRENTLY MARRIED | 7,039.144 40.58 40.58

2. YES, LIVING WITH A PARTNER |494.1966712 2.85 43.43

3. NO, NOT IN UNION | 9,800.0434 56.49 99.92

9. NO RESPONSE | 13.6159936 0.08 100.00

------------------------------+-----------------------------------

Total | 17,347 100.00

-> tabulation of windex5

Wealth |

index |

quintile - |

MICS | Freq. Percent Cum.

------------+-----------------------------------

1. Poorest | 3,035.6179 17.50 17.50

2. Second | 3,217.9453 18.55 36.05

3. Middle | 3,350.3145 19.31 55.36

4. Fourth | 3,859.2053 22.25 77.61

5. Richest | 3,883.9171 22.39 100.00

------------+-----------------------------------

Total | 17,347 100.00

-> tabulation of MVT22B

In the past 12 |

months, felt |

discriminated: |

Sex | Freq. Percent Cum.

---------------+-----------------------------------

1. YES |856.6406846 4.94 4.94

2. NO | 16,447.884 94.82 99.76

8. DK | 41.6715652 0.24 100.00

9. NO RESPONSE | .803324938 0.00 100.00

---------------+-----------------------------------

Total | 17,347 100.00

**WOMEN**

. tab1 DV1A DV1B DV1C DV1D DV1E DV1F WAGE welevel MT2 MT3 MT4 MT9 MT11 HH6 LS1 LS3 LS4 W

> M14 CM1 MA1 windex5 VT22B MSTATUS FN2 [iw=wt]

-> tabulation of DV1A

If she goes |

out with out |

telling |

husband: wife |

beating |

justified | Freq. Percent Cum.

---------------+-----------------------------------

1. YES |5,337.15449 13.75 13.75

2. NO | 33,062.914 85.20 98.95

8. DK | 386.249473 1.00 99.95

9. NO RESPONSE | 19.6821748 0.05 100.00

---------------+-----------------------------------

Total | 38,806 100.00

-> tabulation of DV1B

If she |

neglects the |

children: wife |

beating |

justified | Freq. Percent Cum.

---------------+-----------------------------------

1. YES | 5,838.9281 15.05 15.05

2. NO | 32,626.796 84.08 99.12

8. DK | 319.39353 0.82 99.95

9. NO RESPONSE | 20.8823694 0.05 100.00

---------------+-----------------------------------

Total | 38,806 100.00

-> tabulation of DV1C

If she argues |

with husband: |

wife beating |

justified | Freq. Percent Cum.

---------------+-----------------------------------

1. YES | 5,422.6326 13.97 13.97

2. NO | 33,051.969 85.17 99.15

8. DK | 317.416565 0.82 99.96

9. NO RESPONSE | 13.9813553 0.04 100.00

---------------+-----------------------------------

Total | 38,806 100.00

-> tabulation of DV1D

If she refuses |

sex with |

husband: wife |

beating |

justified | Freq. Percent Cum.

---------------+-----------------------------------

1. YES |7,659.74402 19.74 19.74

2. NO | 30,534.856 78.69 98.42

8. DK | 541.813013 1.40 99.82

9. NO RESPONSE | 69.5869103 0.18 100.00

---------------+-----------------------------------

Total | 38,806 100.00

-> tabulation of DV1E

If she burns |

the food: wife |

beating |

justified | Freq. Percent Cum.

---------------+-----------------------------------

1. YES | 3,143.4364 8.10 8.10

2. NO | 35,384.097 91.18 99.28

8. DK | 256.738578 0.66 99.94

9. NO RESPONSE | 21.7281149 0.06 100.00

---------------+-----------------------------------

Total | 38,806 100.00

-> tabulation of DV1F

If she sleeps |

with another |

man | Freq. Percent Cum.

---------------+-----------------------------------

1. YES |17,508.2344 45.12 45.12

2. NO | 20,481.657 52.78 97.90

8. DK | 721.568066 1.86 99.76

9. NO RESPONSE | 94.5402632 0.24 100.00

---------------+-----------------------------------

Total | 38,806 100.00

-> tabulation of WAGE

Age | Freq. Percent Cum.

------------+-----------------------------------

1. 15-19 |8,498.70872 21.90 21.90

2. 20-24 | 6,322.3846 16.29 38.19

3. 25-29 | 5,953.5023 15.34 53.53

4. 30-34 | 5,309.5624 13.68 67.22

5. 35-39 | 5,230.5974 13.48 80.70

6. 40-44 | 4,209.2244 10.85 91.54

7. 45-49 | 3,282.0203 8.46 100.00

------------+-----------------------------------

Total | 38,806 100.00

-> tabulation of welevel

Education | Freq. Percent Cum.

--------------------+-----------------------------------

0. None | 10,303.101 26.55 26.55

1. Primary | 5,299.9514 13.66 40.21

2. Junior secondary | 3,386.0889 8.73 48.93

3. Senior secondary | 14,164.49 36.50 85.43

4. Higher/tertiary | 5,647.4038 14.55 99.99

9. Missing/DK | 4.96473125 0.01 100.00

--------------------+-----------------------------------

Total | 38,806 100.00

-> tabulation of MT2

Frequency of listening |

to the radio | Freq. Percent Cum.

-------------------------+-----------------------------------

0. NOT AT ALL | 19,361.962 49.89 49.89

1. LESS THAN ONCE A WEEK | 6,072.994 15.65 65.54

2. AT LEAST ONCE A WEEK | 6,917.4628 17.83 83.37

3. ALMOST EVERY DAY | 6,436.3171 16.59 99.96

9. NO RESPONSE |17.26408501 0.04 100.00

-------------------------+-----------------------------------

Total | 38,806 100.00

-> tabulation of MT3

Frequency of watching TV | Freq. Percent Cum.

-------------------------+-----------------------------------

0. NOT AT ALL |18,426.0273 47.48 47.48

1. LESS THAN ONCE A WEEK | 3,764.8794 9.70 57.18

2. AT LEAST ONCE A WEEK | 6,033.5663 15.55 72.73

3. ALMOST EVERY DAY | 10,553.37 27.20 99.93

9. NO RESPONSE | 28.1570731 0.07 100.00

-------------------------+-----------------------------------

Total | 38,806 100.00

-> tabulation of MT4

Ever used a |

computer or a |

tablet | Freq. Percent Cum.

---------------+-----------------------------------

1. YES | 5,192.5648 13.38 13.38

2. NO | 33,520.876 86.38 99.76

9. NO RESPONSE | 92.5596251 0.24 100.00

---------------+-----------------------------------

Total | 38,806 100.00

-> tabulation of MT9

Ever used |

internet | Freq. Percent Cum.

---------------+-----------------------------------

1. YES | 7,428.5419 20.11 20.11

2. NO | 29,407.012 79.63 99.74

9. NO RESPONSE | 95.5441974 0.26 100.00

---------------+-----------------------------------

Total | 36,931.098 100.00

-> tabulation of MT11

Own a mobile |

phone | Freq. Percent Cum.

---------------+-----------------------------------

1. YES |22,568.8738 58.16 58.16

2. NO | 16,214.595 41.78 99.94

9. NO RESPONSE | 22.5310712 0.06 100.00

---------------+-----------------------------------

Total | 38,806 100.00

-> tabulation of HH6

Area | Freq. Percent Cum.

------------+-----------------------------------

1. URBAN |17,805.1701 45.88 45.88

2. RURAL | 21,000.83 54.12 100.00

------------+-----------------------------------

Total | 38,806 100.00

-> tabulation of LS1

Estimation of overall |

happiness | Freq. Percent Cum.

-----------------------------+-----------------------------------

1. VERY HAPPY | 16,450.532 42.39 42.39

2. SOMEWHAT HAPPY | 14,215.34 36.63 79.02

3. NEITHER HAPPY NOR UNHAPPY | 5,335.3611 13.75 92.77

4. SOMEWHAT UNHAPPY | 1,912.9945 4.93 97.70

5. VERY UNHAPPY | 836.900518 2.16 99.86

9. NO RESPONSE | 54.8717285 0.14 100.00

-----------------------------+-----------------------------------

Total | 38,806 100.00

-> tabulation of LS3

Life satisfaction in |

comparison with last |

year | Freq. Percent Cum.

-------------------------+-----------------------------------

1. IMPROVED | 25,239.522 65.04 65.04

2. MORE OR LESS THE SAME | 9,659.6303 24.89 89.93

3. WORSENED | 3,830.9335 9.87 99.80

9. NO RESPONSE | 75.9141923 0.20 100.00

-------------------------+-----------------------------------

Total | 38,806 100.00

-> tabulation of LS4

Life satisfaction |

expectation one year |

from now | Freq. Percent Cum.

-------------------------+-----------------------------------

1. BETTER | 35,648.561 91.86 91.86

2. MORE OR LESS THE SAME | 2,472.1855 6.37 98.23

3. WORSE | 536.475034 1.38 99.62

9. NO RESPONSE | 148.778657 0.38 100.00

-------------------------+-----------------------------------

Total | 38,806 100.00

-> tabulation of WM14

Native language of |

the Respondent | Freq. Percent Cum.

-------------------+-----------------------------------

11. HAUSA | 10,684.15 27.53 27.53

12. IGBO | 5,933.2627 15.29 42.82

13. YORUBA | 6,868.1284 17.70 60.52

14. FULANI | 1,932.7711 4.98 65.50

15. KANURI | 654.30762 1.69 67.19

16. IJAW | 527.732704 1.36 68.55

17. TIV | 923.732729 2.38 70.93

18. IBIBIO | 752.878315 1.94 72.87

19. EDO | 500.031045 1.29 74.16

96. OTHER LANGUAGE | 10,029.005 25.84 100.00

-------------------+-----------------------------------

Total | 38,806 100.00

-> tabulation of CM1

Ever given |

birth | Freq. Percent Cum.

------------+-----------------------------------

1. YES | 25,062.763 64.58 64.58

2. NO |13,743.2367 35.42 100.00

------------+-----------------------------------

Total | 38,806 100.00

-> tabulation of MA1

Currently married or living |

with a man | Freq. Percent Cum.

------------------------------+-----------------------------------

1. YES, CURRENTLY MARRIED | 21,628.81 55.74 55.74

2. YES, LIVING WITH A PARTNER | 2,299.5108 5.93 61.66

3. NO, NOT IN UNION | 14,860.716 38.29 99.96

9. NO RESPONSE | 16.9632131 0.04 100.00

------------------------------+-----------------------------------

Total | 38,806 100.00

-> tabulation of windex5

Wealth |

index |

quintile - |

MICS | Freq. Percent Cum.

------------+-----------------------------------

1. Poorest |6,869.51383 17.70 17.70

2. Second | 7,239.1455 18.65 36.36

3. Middle | 7,561.5863 19.49 55.84

4. Fourth | 8,308.0438 21.41 77.25

5. Richest | 8,827.7105 22.75 100.00

------------+-----------------------------------

Total | 38,806 100.00

-> tabulation of VT22B

In the past 12 |

months, felt |

discriminated: |

Gender | Freq. Percent Cum.

---------------+-----------------------------------

1. YES | 2,325.3991 5.99 5.99

2. NO | 36,353.204 93.68 99.67

8. DK | 120.388977 0.31 99.98

9. NO RESPONSE | 7.00794685 0.02 100.00

---------------+-----------------------------------

Total | 38,806 100.00

-> tabulation of MSTATUS

Marital/Union status of woman | Freq. Percent Cum.

------------------------------+-----------------------------------

1. Currently married/in union | 23,928.32 61.66 61.66

2. Formerly married/in union | 2,068.4795 5.33 66.99

3. Never married/in union | 12,784.861 32.95 99.94

9 | 24.3393654 0.06 100.00

------------------------------+-----------------------------------

Total | 38,806 100.00

-> tabulation of FN2

Do you own a |

bank account | Freq. Percent Cum.

---------------+-----------------------------------

1. YES | 13,720.793 35.36 35.36

2. NO | 25,024.628 64.49 99.84

8. DON?T KNOW | 60.4593164 0.16 100.00

9. NO RESPONSE | .11979191 0.00 100.00

---------------+-----------------------------------

Total | 38,806 100.00
